# Importance of Secondary Prevention in Coronary Heart Disease

**DOI:** 10.3390/medicina61112011

**Published:** 2025-11-10

**Authors:** Svetlana Mosteoru, Nilima Rajpal Kundnani, Andreea Rus, Simona Ilin, Veronica Ciocan, Nicolae Albulescu, Marioara Nicula Neagu, Laura Gaita, Dan Gaita

**Affiliations:** 1 Ph.D. School Department, “Victor Babes” University of Medicine and Pharmacy, 300041 Timisoara, Romania; 2 Research Centre of Timisoara Institute of Cardiovascular Diseases, IBCV-TIM Advanced Research Center, “Victor Babes” University of Medicine and Pharmacy, 3000041 Timisoara, Romania; 3 University Clinic of Internal Medicine and Ambulatory Care, Prevention and Cardiovascular Recovery, Department VI—Cardiology, “Victor Babes” University of Medicine and Pharmacy, 3000041 Timisoara, Romania; 4 Municipality Emergency Hospital, 300172 Timisoara, Romania; 5 Department of Forensic Medicine, Bioethics, Medical Ethics and Medical Law, Center for Ethics in Human Genetic Identifications, “Victor Babes” University of Medicine and Pharmacy, 3000041 Timisoara, Romania; 6 Division of Internal Medicine II—Cardiology, “Victor Babes” University of Medicine and Pharmacy, 3000041 Timisoara, Romania; 7 Centre for Molecular Research in Nephrology and Vascular Disease, “Victor Babes” University of Medicine and Pharmacy, 3000041 Timisoara, Romania; 8 “Pius Brînzeu” Emergency County Hospital, 300723 Timisoara, Romania; 9 Physiology Discipline, Faculty of Bioengineering of Animal Resources, University of Life Sciences “King Mihai I” from Timisoara, 300645 Timisoara, Romania; 10 Department of Internal Medicine II, “Victor Babes” University of Medicine and Pharmacy, 300041 Timisoara, Romania

**Keywords:** coronary heart disease, secondary prevention, hypertension, LDL, HbA1c

## Abstract

*Background and Objectives*: The present study evaluates documentation and control of cardiovascular risk factors (RFs) in patients with coronary heart disease (CHD) during routine outpatient visits at a single tertiary center in western Romania and places these findings in descriptive context relative to SURF-CHD reports from Europe. *Materials and Methods*: We have enrolled 136 consecutive patients between 18 and 80 years old with coronary artery disease attending routine outpatient clinic check-ups between May 2019 and July 2020. All patients had been diagnosed with acute coronary syndrome or stable angina pectoris and had been treated either by PCI or CABG. Comparisons with SURF-CHD were primarily descriptive due to non-harmonized denominators and lack of patient-level data; inferential testing was limited to variables with clear n/N in both cohorts. *Results*: Most patients (81%) were males with a mean age of 61.7 years. 93.4% of the patients had undergone PCI, and 4.4% had coronary artery bypass graft (CABG). Regarding risk factors, 25% were current smokers, while 50% were former smokers and the mean BMI value was 29.9 (±6.07). While most patients (80.1%) revealed no previous history of dyslipidemia, 62.5% had no previous history of arterial hypertension, and 84.6% had no previous history of diabetes mellitus. Mean LDL cholesterol levels after a major coronary event remained 93.55 (±43.52) mg/dL, mean HbA1c levels were 7.86 (±1.40)%, while mean systolic blood pressure was 129 (±14.9) mmHg. *Conclusions*: In this single-center audit, several modifiable RFs remained suboptimally controlled despite established CHD. These results should not be generalized nationally; rather, they highlight center-level opportunities for improving secondary prevention and underscore the need for multicenter, nationally representative registries in Romania.

## 1. Introduction

European Action on Secondary and Primary Prevention by Intervention to Reduce Events was a series of repeated surveys spanning between 1995 and 2018 under the auspices of the ESC. Meanwhile, SUrvey of Risk Factors in Coronary Heart Disease (SURF CHD), developed in collaboration with the European Association of Preventive Cardiology, was created as a straightforward audit tool for use during routine clinic visits to evaluate risk factors and assess adherence to clinical guidelines in everyday practice. Romania has been an active participant in this survey, along with 29 other countries around the world, and data from our national cohort will be analyzed in this study [1].

The objective of this study is to assess the documentation of cardiovascular risk factors (RFs) in patients with coronary heart disease (CHD) during routine clinical evaluations. It also aims to analyze the management strategies the achievement of RF and lifestyle modification goals. Ultimately, the study seeks to provide an objective and comprehensive evaluation of risk factor recording and control across a wide patient population, while offering valuable insights by comparing Romanian patients to the European population.

## 2. Material and Methods

### 2.1. Patient Recruitment

We consecutively enrolled adults aged 18–80 years with established CHD who attended routine outpatient follow-up visits at the Institute for Cardiovascular Diseases Timișoara between May 2019 and July 2020. Eligible patients had a prior diagnosis of acute coronary syndrome or stable angina and had undergone PCI or CABG according to ESC guidelines. Patients who declined the use of anonymized data were excluded; no other selection criteria were applied. This study reflects a single-center experience in a predominantly urban, western Romanian setting. Ethical approval for the central survey was waived by the Medical Ethics Committee of the University Medical Center Utrecht (protocol number 17/534). Ethical approval was obtained or waived in individual participating centers prior to participation. In our center, ethical approval was granted under an exemption due to the retrospective analysis of anonymized clinical data, in accordance with national regulations and the Helsinki Declaration (Article 23) by the Ethics Committee of the Institute for Cardiovascular Diseases Timisoara.

### 2.2. Data Collection

Data collected included demographic characteristics such as gender, age and race, as well as smoking status (ex-smoker was defined as more than six months of abstinence). Physical activity (considered as ’moderate’ with a physical activity of 30 min, 3–5 times per week), family history of premature cardiovascular disease (positive if there is a diagnosis of stroke, CHD or peripheral vascular disease before the age of 55 in male and 65 in female first-degree relatives), and education level. Medical history was also recorded, including data about dyslipidemia and diabetes mellitus, as well as information regarding participation in cardiac rehabilitation programs. Height, weight, waist circumference, heart rate, and blood pressure (BP) were recorded on the day of the clinic visit. Laboratory analyses were recently drawn before the current visit as part of the local standard of care. They included fasting total cholesterol (TC), fasting LDL-Cholesterol, fasting HDL-Cholesterol, fasting triglycerides (TG), fasting glucose and HbA1c if the patient was diabetic.

### 2.3. Statistical Analysis

Data was stored and managed using Microsoft Excel 2010, and statistical analyses were performed using MedCalc 23.3.7. Mean and standard deviations expressed to two decimal places were used for scale-type data, and group percentage was used for ordinal data. Regarding risk-factor target attainment denominators, for each target, percentages were calculated using within-cohort denominators: LDL-C target among patients with an available LDL-C value; HbA1c target among patients with diabetes and an available HbA1c; blood pressure target among patients with an in-visit BP measurement; BMI and waist circumference among patients with those measurements recorded. For SURF-CHD comparators, published European summaries lacked complete or harmonized denominators for these targets; therefore, we did not perform inferential Romania-vs-Europe testing for target attainment and present any European figures descriptively. Between-cohort tests were performed only where both numerators and denominators were unambiguous in Romania and Europe; risk-factor target attainment is therefore reported descriptively. Exact Romanian denominators (n/N) for each target are provided in Supplementary Table S1 and in figure footnotes. This aligns with the limitation acknowledged regarding missingness in SURF denominators.

## 3. Results

**Figure 1:**
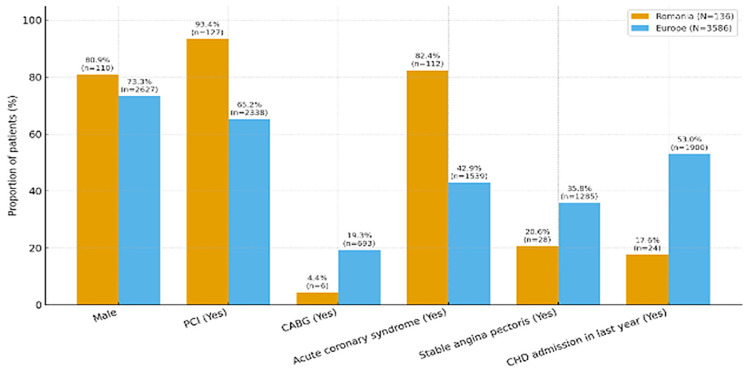
Comparison of patient characteristics between Romanian and European study groups. Compared with the European SURF cohort, our center’s patients were younger and more often admitted with ACS, with substantially higher PCI and lower CABG rates. These differences likely reflect center-level practice patterns and case mix rather than national trends and should be interpreted within a single-center context.

Most patients (81%) were males with a mean age of 61.7 years. 93.4% of the patients had undergone PCI and 4.4% had coronary artery bypass graft (CABG), as compared to the rest of Europe, where 65.2% had PCI and 19.3% had CABG. However, only 17.6% of the patients had had a hospital re-admission in the last year due to coronary heart disease, as opposed to more than half (53%) in the rest of Europe (Table 1) (Figure 1).

**Figure 2:**
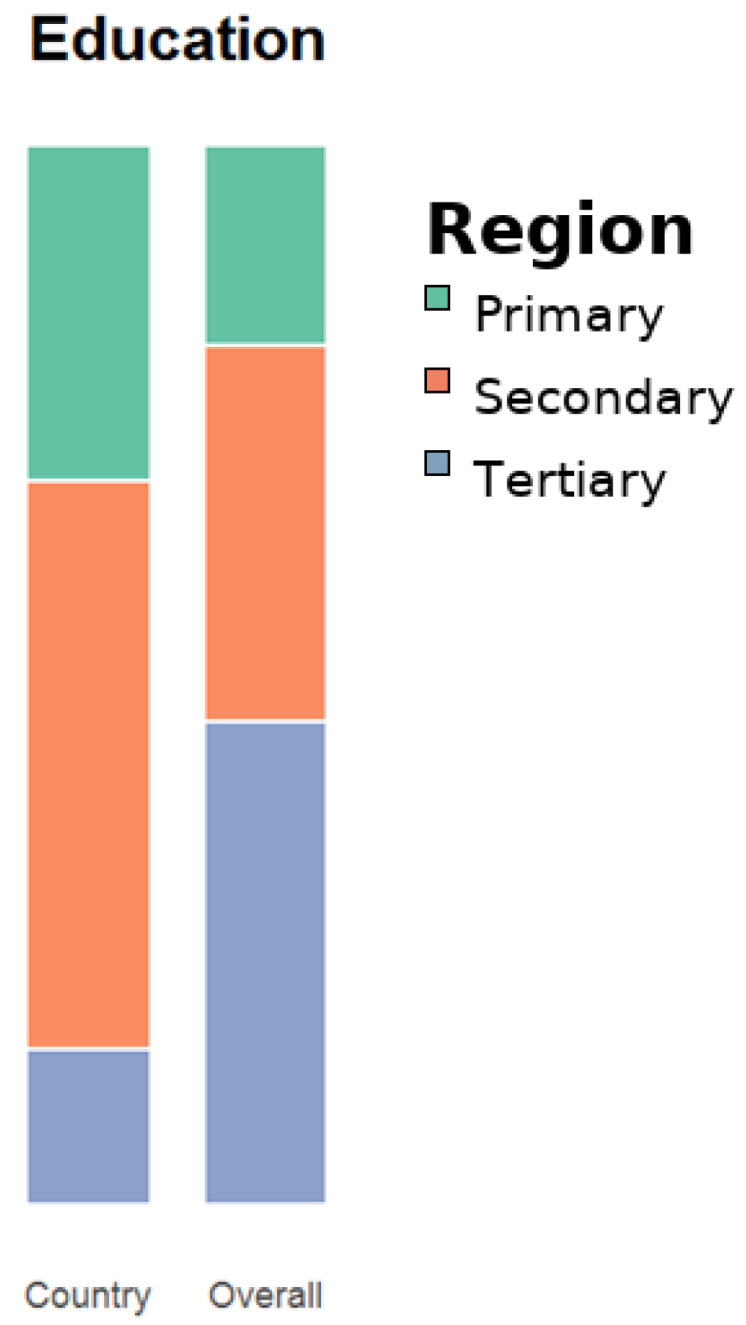
Education level. The cohort skews toward secondary education, and higher body weight is more frequent in lower education strata, consistent with socio-educational gradients reported elsewhere. As this is a single-center, largely urban sample, these gradients may not mirror rural populations. Distribution by highest completed education in the Romanian cohort (N = 136). Values are %.

**Figure 3:**
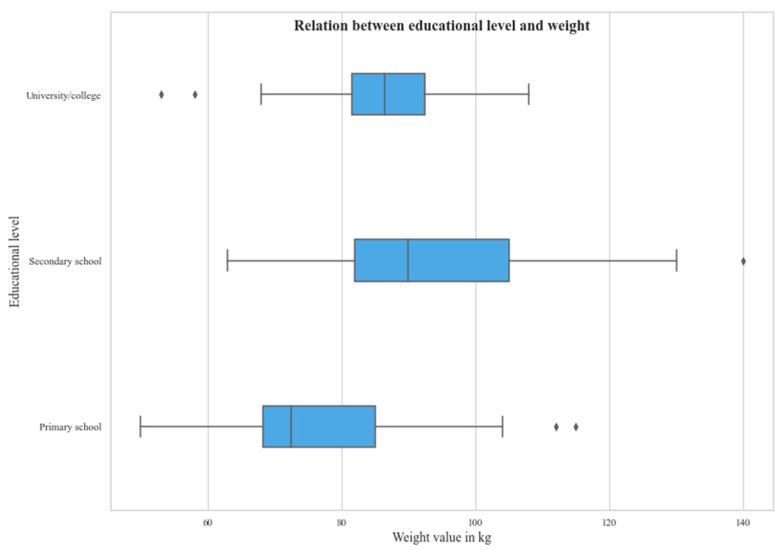
Weight differences among education level. Body weight (kg) across education strata. Units: kg.

More than half of the patients had secondary education (54%), while only 31% had undergone tertiary education (Figure 2). 40% of patients were obese. When comparing education level to the weight of participants there was a statistically significant difference (Figure 3).

**Figure 4:**
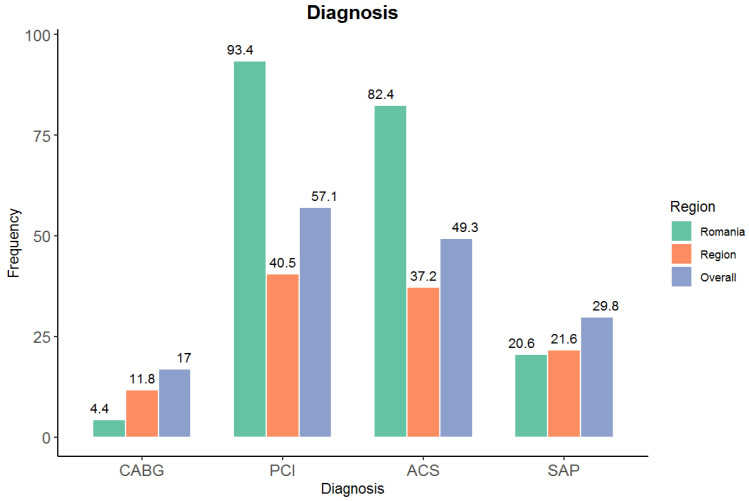
Comparison of inclusion diagnosis between Romania, the region (Europe) and overall (worldwide) expressed as percentage. The predominance of ACS at presentation in our clinic suggests a referral pattern favoring acute presentations. This may contribute to the observed preference for PCI and should not be generalized to other Romanian regions or levels of care. Acute coronary syndrome (ACS) and stable angina pectoris (SAP) at inclusion, shown as % of cohort. Denominators: Romania N = 136; Europe N = 3586.

In Romania, most patients had been admitted due to an acute coronary syndrome (82.4%) and only 20.6% due to stable angina, as compared to 37.2% for an acute coronary syndrome in Europe and 21.6% for stable angina (Figure 4).

Although only 26% of patients had a family history of premature cardiovascular disease, almost half of them (48%) had a previous history of cardiovascular disease. 37.5% were already diagnosed with arterial hypertension, 19.9% had dyslipidemia and 15.4% diabetes mellitus. Only 25% of these patients had never smoked, while 50% were ex-smokers and 25% current smokers. However, when considering multiple risk factors, 22.16% had no risk factors, 60.44% had one risk factor, 32.24% had two risk factors, 17.12% had three risk factors and 5.4% had four risk factors.

68% of patients claimed to do moderate physical activity (30 min, 3–5×/week), while only 12% did more than 30 min. Mean systolic blood pressure was 129 mmHg, lower than for Europe (133 mmHg) or worldwide (132 mmHg). The same pattern can be observed with mean diastolic blood pressure 76.8 mmHg, as compared to 78.8 mmHg in Europe and 78 mmHg worldwide. Mean heart rate however, was similar between Romania and the rest of Europe (71.4 bpm) and lower than worldwide (73.2 bpm).

**Figure 5:**
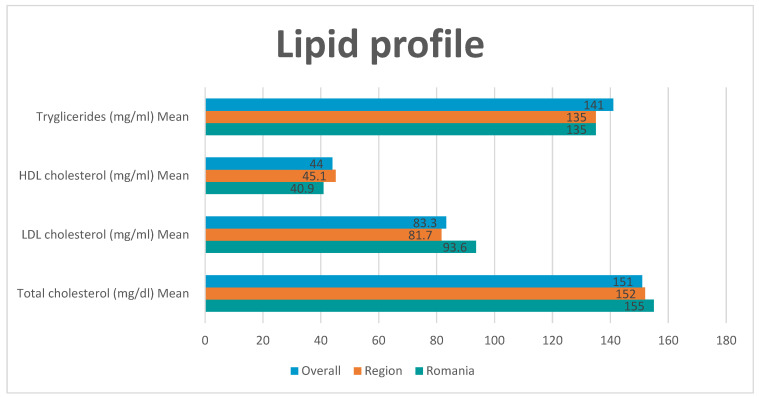
Lipid profile. Mean LDL-C and total cholesterol were higher in our cohort than region-wide descriptive reports, consistent with the low proportion meeting LDL targets. This identifies a clear target for intensifying lipid-lowering therapy and follow-up. Mean (SD) LDL-C, total cholesterol, HDL-C, and triglycerides in mg/dL. (SI: mmol/L = mg/dL ÷ 38.67 for cholesterol fractions; ÷ 88.57 for triglycerides).

When it comes to the lipid profile both mean LDL cholesterol and mean total cholesterol are higher in Romania as compared to the rest of the region and worldwide (93.6 mg/dL versus 81.7 mg/dL and 83.3 mg/dL for LDL cholesterol and 155 mg/dL versus 152 mg/dL and 151 mg/dL for total cholesterol). Only mean triglyceride levels were similar between Romania and Europe (135 mg/dL) and lower than worldwide (141 mg/dL). (Figure 5)

On the other hand, mean HbA1c levels were lower in Romania (7.86%) as compared to Europe (8.64%) and worldwide (9.02%).

**Figure 6:**
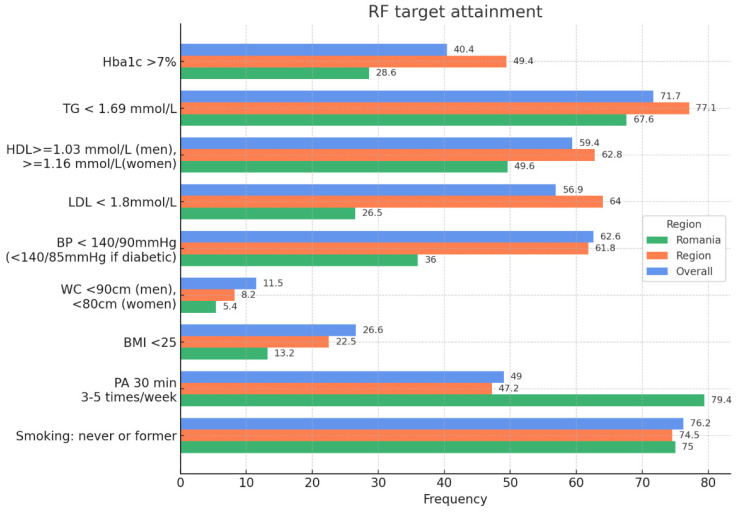
Risk factors target attainment. Within our cohort, attainment was lowest for BMI and waist circumference targets and modest for LDL-C and blood pressure. We now report explicit denominators (n/N) for each target in Supplementary Table S1 and footnotes to Figure 6. Because SURF-CHD European denominators are incomplete/heterogeneous, we present European values descriptively only and avoid inferential comparisons for these outcomes. Targets: non-smoking; physical activity ≥ 30 min, 3–5×/week; BMI < 25 kg/m^2^; waist circumference < 94 cm (men), <80 cm (women); BP < 140/90 mmHg (or <140/85 mmHg if diabetes); LDL-C < 70 mg/dL (1.8 mmol/L); HbA1c < 7% in diabetics.

However, when it comes to risk factors target attainment our cohort has a slightly higher percentage of non-smokers or former smokers (75%) as compared to Europe (74.5%), but lower than worldwide (76.2%). Almost 80% of the patients were doing regular physical activity for approximately 30 min, 3–5 times per week, as compared to 47.2% in Europe and 49% worldwide. (Figure 6)

Nevertheless, BMI below 25 kg/m^2^ is only achieved by 13.2% of the patients as compared to 22.5% in Europe and 26.6% worldwide. A similar pattern was observed in regard to blood pressure, where only 36% of the patients from our cohort achieved the target below 140/90 mmHg or below 140/85 if diabetic, while 61.8% had achieved this target in Europe and 62.6% worldwide. The same holds true for achieving LDL cholesterol below 1.8 mmol/L (70 mg/dL), only 26.5% of the included subjects reached this target in our cohort, as compared to 64% in Europe. Similarly, only 28.6% of subjects reached the HbA1c target below 7% in Romania, while almost half of the subjects in Europe did (49.4%), and regarding waist circumference, only 5,4% managed to attain the target. (Figure 6)

In order to compare statistically, not just descriptively, the values found in our cohort in Romania to the ones reported in the SURF-CHD study, we performed risk differences, risk ratios, odds ratios and z-tests for categorical variables, and a Welch *t*-test for age (a continuous variable). We should note at this point a limitation, which is the fact that we could not test risk-factor target attainment since SURF-CHD had missingness and diabetic-only denominators. Moreover, we unfortunately did not have access to patient-level data or exact SURF counts for Europe with known denominators. More on this will be elaborated in the Discussions section.

Below are the values we found for comparative inferential tests (Table 2) and the Welch *t*-test for age (Table 3).

- Male sex: higher in Romania (80.9%) than Europe (73.3%); *p* = 0.048
- CABG (Yes): lower in Romania (4.4%) vs. Europe (19.3%); *p* < 0.001.
- PCI (Yes): higher in Romania (93.4%) vs. Europe (65.2%); *p* < 0.001.
- Acute coronary syndrome (Yes): higher in Romania (82.4%) vs. Europe (42.9%); *p* < 0.001.
- Stable angina (Yes): lower in Romania (20.6%) vs. Europe (35.8%); *p* < 0.001.
- CHD readmission last year (Yes): lower in Romania (17.6%) vs. Europe (53.7% of non-missing); *p* < 0.001.
- Age: Romania mean 61.7 vs. Europe 64.9 years; Welch *t*-test *p* < 1 × 10^−6^ (younger in Romania).

## 4. Discussion

This analysis reflects care delivered at a single tertiary center in western Romania and should be interpreted as a center-level audit rather than a nationally representative estimate. Patterns observed here (e.g., younger age, ACS predominance, higher PCI share) may reflect local referral and revascularization practices and cannot be generalized to rural or other regional settings without multicenter data. In this clinical audit, the primary aim was to assess the management of modifiable cardiovascular risk factors (RFs) and the application of medical therapies for secondary prevention in patients with established coronary heart disease (CHD). Evidence suggests that, despite a history of CHD, these modifiable RFs remain inadequately controlled in many cases [2,3]. A significant discrepancy persists between evidence-based guideline recommendations and actual clinical practice [4]. While guidelines emphasize the importance of achieving targets for RFs to reduce cardiovascular risk and prevent further progression of CHD [5], these objectives are frequently not met in practice. This shortfall appears to be influenced not only by the nature and frequency of clinical follow-up but also by patients’ understanding of their condition and their adherence to therapeutic and lifestyle recommendations [6].

Smoking remains the leading cause of preventable mortality worldwide [7]. In the current study, smoking prevalence was observed in 25% of the analyzed cohort, while 50% were identified as former smokers, and the remaining 25% had never smoked. Data from a recent cross-sectional analysis in the United States, which included adults aged 18 years and older, demonstrated that despite increased receipt of professional advice to quit smoking, cessation rates remained suboptimal in certain subgroups. These included younger adults, individuals lacking health insurance, ethnic minority populations, and those without additional smoking-related comorbidities [8]. Potential interventions to improve smoking cessation could be immediate bedside counseling and follow-up nurse-led brief interventions either by telephone or during routine clinic visits.

Insufficient physical activity represents a significant modifiable risk factor for coronary heart disease (CHD) [9]. Current World Health Organization (WHO) guidelines recommend that all adults should engage in at least 150–300 min of moderate-intensity physical activity or 75–150 min of vigorous-intensity activity on a weekly basis [10]. Nevertheless, only approximately one in four adults (27.5%) meet these guidelines [11]. In our study around 12% did more than 30 min of physical activity 3–5 times per week. Importantly, even small increments in physical activity or replacing sedentary behaviors with light-intensity exercises (such as using stairs instead of elevators) have been shown to reduce cardiovascular risk and positively impact health outcomes [9,12]. In the present study, 68% of participants reported engaging in moderate-intensity physical activity for at least 30 min, 3–5 times per week, whereas 20% reported performing less than 30 min of moderate activity. Implementing programs that integrate physical activity into daily routines—such as physical education in schools and access to exercise facilities in work environments—can facilitate adherence and encourage regular participation. mHealth which is marked out by the use of personal digital instrumentals such as phones and wireless devices for individualized health could serve as an instrument for improving physical activity and cardiac rehabilitation [13].

Moreso, a systematic review and meta-analysis showed that mobile health applications significantly reduce the risk of unplanned hospital readmissions in patients with recent MI/PCI. Smartphone-based interventions for secondary prevention after MI or PCI represent a promising approach to improving post-MI and post-PCI care and should be integrated into routine cardiovascular follow-up [14].

Obesity is a widespread and escalating public health challenge worldwide [15]. It is not only a significant risk factor for coronary heart disease (CHD), but it also contributes to the development of associated conditions such as dyslipidemia, arterial hypertension, and type 2 diabetes mellitus [16]. Evidence from randomized clinical trials indicates that intentional weight reduction through structured programs, can substantially reduce cardiovascular risk and mortality in patients with established CHD [17]. In our study population, only 12% had a normal body mass index (BMI), while 48% were classified as overweight and 40% as obese. The median BMI in the study cohort was 29.6 kg/m^2^, with no significant differences observed between men and women (*p* < 0.05). Despite the high prevalence of overweight and obesity, none of the participants engaged in a CR program. Participation in CR following a CAD event has been consistently shown to significantly improve cardiovascular outcomes and reduce overall mortality [18]. We also believe nutrition advice should be offered upon discharge, even considering the prescription of anti-obesity medication in certain cases.

We have analyzed the prevalence of previously diagnosed risk factors such as arterial hypertension (37.5%), dyslipidemia (19.9%) and diabetes mellitus (15.4%). 52% reported to not have a previous medical history of any of the diseases that were mentioned above.

There has been a persistent global increase in the prevalence of type 2 diabetes mellitus, currently affecting approximately 8.8% of the world’s population [19]. Individuals with DM II have a 2- to 4-fold increased risk of developing cardiovascular diseases compared to those without diabetes [20]. Cardiovascular risk and associated mortality are directly correlated with increasing levels of glycated hemoglobin (HbA1c). Specifically, the lowest mortality rates are observed with HbA1c levels between 6–6.9%, while levels ≥ 7% are associated with increased cardiovascular and overall mortality [21]. Consequently, comprehensive management of cardiovascular risk factors (RFs) and pharmacological strategies to achieve optimal glycemic control are crucial in preventing the progression of CVD and in reducing related mortality [22]. Notably, diabetic patients have an 18% increased risk of cardiovascular mortality and a 16% increase in all-cause mortality [23].

The UK Prospective Diabetes Study (UKPDS) reported that while intensive glycemic control did not result in a short-term reduction of cardiovascular events, significant reductions were observed after 10–17 years of sustained control [24].

Early screening for dysglycemia could help improve outcomes for these patients, while considering immediate initiation of new anti-diabetic classes for patients already diagnosed with DM.

Arterial hypertension, previously diagnosed in 37.5% of the patients in this study, represents a significant risk factor for cardiovascular disease. The association between cardiovascular risk and elevated blood pressure depends not only on the degree of hypertension but also on its duration [25]. A comprehensive meta-analysis has demonstrated that an incremental increase in systolic blood pressure is associated with a proportional rise in cardiovascular mortality, underscoring the importance of achieving optimal blood pressure control [26]. Consequently, effective management of arterial hypertension is vital in reducing both cardiovascular risk and all-cause mortality.

For patients with established arterial hypertension, regular blood pressure monitoring is strongly recommended to evaluate treatment response and to guide therapeutic adjustments when necessary. Effective management involves not only pharmacological antihypertensive therapy but also lifestyle interventions, such as dietary modifications, weight management, regular physical activity, and smoking cessation, to achieve and maintain optimal blood pressure targets [27,28].

Although only 19.8% of the patients in the study group were previously diagnosed with dyslipidemia, actually 89.6% exhibited at least one abnormality in their lipid profile, out of which only 26.5% reached their LDL cholesterol target below 70 mg/dL. Aggressive lipid escalation therapy could be crucial in attaining risk factor control in such patients.

Our inferential analyses demonstrated that CHD patients from our cohort are significantly younger, more likely to present with acute coronary syndromes, and disproportionately steer toward PCI rather than CABG. This contrasts with European patterns and shows potential fundamental differences in clinical practice.

### Limitations of the Study

Several limitations should be acknowledged when interpreting our findings. First, although we were able to perform inferential statistical comparisons between the our cohort and the larger European SURF CHD registry, these analyses were conducted on aggregated data rather than patient-level datasets. This meant that we could only calculate risk differences, risk ratios, odds ratios, and Welch’s *t*-tests on variables with clearly defined denominators in both cohorts (e.g., sex, age, PCI/CABG rates, admission diagnosis, hospital readmissions). For other clinically relevant endpoints, such as risk factor target attainment (LDL, HbA1c, waist circumference, blood pressure), comparisons were limited by missingness and a lack of exact counts in the published European registry. As a result, we could not perform robust inferential statistics for these outcomes and our conclusions in these domains remain descriptive.

Our single-center Romanian sample size was relatively small (N = 136) compared to the European comparator cohort (N = 3586). While statistically significant differences emerged for many variables, the wide confidence intervals for some estimates indicate that precision is limited. Thus, it is difficult to generalize the results. We therefore call for the need of larger national registries to validate these observations.

Another limitation of this study is the single-center nature of our survey, which has taken place in the one center from a rather well-developed area of the country, making it difficult to expand the results to the entire country. More centers should be included in future surveys in order to improve national representativity.

The cross-sectional nature of our data collection restricts our ability to capture longitudinal improvements in risk factor control or outcomes after revascularization. For example, although our patients were younger and more likely to present with ACS, we cannot comment on how these differences impact long-term prognosis, recurrent events, or survival compared to European patients.

## 5. Conclusions

This clinical audit was conducted to assess the management of modifiable risk factors for secondary prevention in patients with established coronary heart disease. Although CHD is a common finding in adults, the study revealed that the modifiable risk factors remained suboptimally controlled in our cohort, highlighting a substantial discrepancy between clinical guideline recommendations and current practice. There is no possibility to change the non-modifiable risk factors, but modifiable risk factors, if controlled even after the appearance of the disease, can help achieve a better prognosis.

These findings highlight the crucial need for enhanced patient education and engagement to promote improved awareness and self-management of these risk factors. Prevention is always better than a cure; hence, primary health care physicians and cardiologists should both actively participate in combining medical expertise with patient-centered counseling and follow-up. They can empower patients to take control of modifiable risk factors, significantly reducing their risk of developing cardiovascular disease. The new strategy for cardiovascular prevention recently published by the Romanian Ministry of Health could be the key to implementing these strategies for reducing the cardiovascular disease burden.

These results should not be generalized nationally; rather, they highlight center-level opportunities for improving secondary prevention and underscore the need for multicenter, nationally representative registries in Romania.

## Figures and Tables

**Table 1:** Comparison of patient characteristics between Romanian and European study groups.

	Romania	Europe
	(N = 136)	(N = 3586)
Male	110 (80.9%)	2627 (73.3%)
Mean Age (SD)	61.7 (10.9)	64.9 (10.7)
Median [Min, Max]	63.0 [28.0, 84.0]	65.0 [27.0, 99.0]
CABG	6 (4.4%)	693 (19.3%)
PCI	127 (93.4%)	2338 (65.2%)
Acute Coronary Syndrome	112 (82.4%)	1539 (42.9%)
Stable Angina Pectoris	28 (20.6%)	1285 (35.8%)
CHD hospital admission in the last year		
Yes	24 (17.6%)	1900 (53.0%)
Unknown	0 (0%)	47 (1.3%)
		3 (0.1%)

CHD coronary heart disease.

**Table 2:** Romania vs. Europe inferential tests. Two-proportion z-test (pooled variance); two-sided *p*-values. CIs for RD are Wald; CIs for RR/OR are log-Wald.

Variable	Romania n/N (%)	Europe n/N (%)	Risk Difference (95% CI)	Risk Ratio (95% CI)	Odds Ratio (95% CI)	z	*p*
Male sex	110/136 (80.9%)	2627/3585 (73.3%)	7.6% [0.1%, 15.2%]	1.10 [1.01, 1.20]	1.54 [1.00, 2.38]	1.97	0.048
CABG (Yes)	6/136 (4.4%)	693/3586 (19.3%)	−14.9% [−21.6%, −8.2%]	0.23 [0.10, 0.50]	0.19 [0.08, 0.44]	−4.37	1.24 × 10^−5^
PCI (Yes)	127/136 (93.4%)	2338/3586 (65.2%)	28.2% [20.1%, 36.3%]	1.43 [1.36, 1.51]	7.53 [3.82, 14.86]	6.82	8.99 × 10^−12^
Acute Coronary Syndrome (Yes)	112/136 (82.4%)	1539/3586 (42.9%)	39.4% [30.9%, 47.9%]	1.92 [1.76, 2.09]	6.21 [3.97, 9.69]	9.09	1.02 × 10^−19^
Stable Angina Pectoris (Yes)	28/136 (20.6%)	1285/3585 (35.8%)	−15.2% [−23.4%, −7.1%]	0.57 [0.41, 0.80]	0.46 [0.30, 0.71]	−3.65	2.58 × 10^−4^
CHD admission last year (Yes)	24/136 (17.6%)	1900/3536 (53.7%)	−36.1% [−44.6%, −27.5%]	0.33 [0.23, 0.47]	0.18 [0.12, 0.29]	−8.27	1.35 × 10^−16^

**Table 3:** Age comparison (Welch *t*-test, Romania vs. Europe). Welch’s *t*-test (unequal variances), two-sided *p*-value.

Variable	Romania Mean (SD)	Europe Mean (SD)	Mean Difference (RO-EU)	Welch t	df (Approx)	*p*-Value
Age (years)	61.7 (10.9)	64.9 (10.7)	−3.2	−3.36	~145	9.9 × 10^−4^

## Data Availability

The original contributions presented in this study are included in the article. Further inquiries can be directed to the corresponding authors.

## Supplementary Materials

The following supporting information can be downloaded at https://www.mdpi.com/article/10.3390/medicina61112011/s1: Table S1a: Risk-factor target attainment (Romanian cohort). Table S1b. Measurement availability/missingness (Romanian cohort).
