# Supplementary material for: Importance of Secondary Prevention in Coronary Heart Disease
Source: Medicina (Kaunas). 2025 Nov 10;61(11):2011. doi: 10.3390/medicina61112011 (PMC12654057; doi:10.3390/medicina61112011)
Supplement: Supplementary file 1 [file medicina-61-02011-s001.zip › medicina-3913898-supplementary.pdf]

Supplementary Table S1a. Risk-factor target attainment (Romanian cohort).
Percentages are computed among patients with data available for each target (denominators shown as N). European SURF figures are presented descriptively only in the main text; no inferential RO–EU testing was performed for these outcomes due to incomplete/heterogeneous denominators in published SURF summaries.

Target	Threshold / Definition	Numerator n (meeting target)	Denominator N (with data)	n/N (%)
Non-smoking	Current smoking = No	102	136	75.0%
Physical activity	≥30 min, 3–5×/week (self-reported)*	108	136	79.4%
BMI	<25 kg/m²	18	136	13.2%
Waist circumference (men)	<94 cm	13	110	11.8%
Waist circumference (women)	<80 cm	0	26	0.0%
Blood pressure	<140/90 mmHg	87	136	64.0%
LDL-cholesterol	<70 mg/dL (1.8 mmol/L)	36	136	26.5%
HbA1c (diabetes only)	<7% (among those with HbA1c)	2	7	28.6%
Triglycerides (optional)	No target; report if used		136	

Notes (S1): N equals the number of patients with the relevant measurement (e.g., LDL-C value present; in-visit BP recorded; diabetics with an HbA1c).
*Physical activity counted both “Moderate 30 min 3–5×/week” and “More than above (>30 mins)” as meeting the ≥30 min threshold.

Supplementary Table S1b. Measurement availability / missingness (Romanian cohort).
Counts of patients with vs without data for each variable listed in Fig. 5–6 and the text, to document HbA1c/BP/TG availability as requested by the reviewer.

Variable	Count with data (N)	Missing (N)
LDL-cholesterol measured	136	0
Total cholesterol measured	135	1
HDL-cholesterol measured	135	1
Triglycerides measured	136	0

Variable	Count with data (N)	Missing (N)
In-visit blood pressure recorded	136	0
HbA1c measured (in diabetics)	7	
BMI recorded	136	0
Waist circumference recorded	136	0
Smoking status recorded	136	0
Physical activity recorded	136	0

Footnote (S1b): Denominators in S1 use the “Count with data (N)” column above (e.g., LDL-C target among those with an LDL-C value; HbA1c target among diabetics with HbA1c). “Missing (N)” for HbA1c is blank because the dataset did not include a diabetes denominator.
